# Maternal morbidity: Time for reflection, recognition, and action

**DOI:** 10.1002/ijgo.12499

**Published:** 2018-05-23

**Authors:** Lale Say, Doris Chou, the WHO Maternal Morbidity Working Group (MMWG), Kelli Barbour, Maria Barreix, Jose G. Cecatti, Maria L. Costa, Sara Cottler, Olubukola Fawole, Veronique Filippi, Tabassum Firoz, Luis Gadama, Atf Ghérissi, Gathari N. Gichuhi, Gill Gyte, Michelle Hindin, Anoma Jayathilaka, Amanda Kalamar, Yacouba Kone, Nenad Kostanjsek, Isabelle Lange, Laura A. Magee, Arvind Mathur, Affette McCaw‐Binns, Mark Morgan, Stephen Munjanja, Max Petzold, Elizabeth Sullivan, Frank Taulo, Özge Tunçalp, Rachel Vanderkruik, Peter von Dadelszen

**Affiliations:** ^1^ UNDP–UNFPA–UNICEF–WHO–World Bank Special Programme of Research Development and Research Training in Human Reproduction (HRP) Department of Reproductive Health and Research WHO Geneva Switzerland

**Keywords:** Maternal morbidity

Efforts to improve maternal health globally are often viewed simply as measures to avoid maternal death. While declining mortality can be a useful proxy measure for improved health when it comes to setting goals in line with the global sustainable development agenda, it is doubtful that any woman, mother, family member, or community considers “good maternal health” to mean simply surviving pregnancy and childbirth. How women *experience* pregnancy and childbirth is rarely documented or discussed by policy makers, program managers, or healthcare providers, nor is it commonly reflected upon by the woman's family or possibly even herself. Possible reasons for this lack of consideration—and lack of even a common understanding of “well‐being” during pregnancy, labor, childbirth, and in the immediate postpartum period—could be that pregnancy and childbirth are accepted as transitory life events that are not as salient as a death or a severe complication, or that as “experiences” they are too difficult to describe, quantify, or analyze. Yet, given the opportunity, almost every person and community has a story to tell about pregnancy and childbirth, from their own personal experience or those of their relatives, friends, or fellow community members.

The quantifiable aspects of these “stories” are occasionally described in the literature, such as the often‐quoted statistic that there are 20–30 cases of morbidity for every maternal death,^1^, ^2^ and thematic narrative summaries have been provided on this topic in the reports of some qualitative ethnographic studies. Quantitative descriptions that compare binary assessments of morbidity (i.e. “yes/some” versus “no” morbidity) may not be sufficient for assessment of maternal morbidity. Perhaps a complementary and more holistic approach—which acknowledges the combined influence/impact of the woman's own experiences, her environment, and current biomedical knowledge/technology—could shed more light on the experience of maternal health and well‐being.

In 2012, WHO initiated a five‐year project, funded by the Bill & Melinda Gates Foundation, with the aim of developing the evidence base on maternal morbidity through improving the scientific basis for defining, measuring (and estimating), and monitoring it. A multidisciplinary group was convened including academics, clinicians, and public health program managers from six continents and a variety of settings, bringing together their cumulative knowledge and expertise. The dedicated members of this collaboration—the Maternal Morbidity Working Group (MMWG)—systematically unpacked the meaning of maternal morbidity, and examined in depth how best to define, describe, and measure it for the purposes of research, epidemiology, and ultimately to improve women's experience of the care they receive.

Throughout the process, the aim was to close the gap between measuring morbidity for programmatic purposes and assessing its actual impact on a woman's life (including describing the experience of it)—the aspect that had previously been neglected. The definition for maternal morbidity that the MMWG eventually arrived at was: “any health condition attributed to and/or complicating pregnancy and childbirth that has a negative impact on the woman's well‐being and/or functioning”.^3^ This definition allows for conditions to be understood from a woman's point of view and assessed in terms of how they affect her life. Next, keeping this definition firmly in mind, the challenge was to establish how maternal morbidity could be meaningfully and consistently measured at the healthcare facility and community level across varying country and regional settings. Beyond establishing the burden of disease, would the approach be able to document the issues that are important to women themselves?

The group's early discussions focused on identifying the starting point for this body of work. It was decided that, to facilitate the necessary innovative thinking, each expert member should discard their own notions about maternal morbidity and think instead from the woman's perspective, starting by asking a basic, yet surprisingly bold, question: “what was the woman's lived experience?” Initial answers to this question are offered in a qualitative review by Lange et al. (unpublished data, March 2018), which describes how women in low‐ and middle‐income countries experience maternal morbidity; the findings of this paper informed, influenced, and shaped the MMWG's discussions and decisions. Lange et al.'s synthesis of 47 articles encompassed the views of women from Sub‐Saharan Africa, South Asia, Southeast Asia, and one Latin American country, describing the implications of a range of morbidities on women's lives, highlighting the strong links between their physical bodies and the social perceptions of their illnesses. Some of the key conclusions included: morbidities and women living with them were stigmatized; morbidities had negative consequences for women's financial situations and their ability to support themselves; poor physical health often led to pain, discomfort, and feelings of estrangement; and precarious emotional and psychological health exacerbated by severe and nonsevere morbidities could lead to depression and anxiety.

As Lange et al. describe, the overlapping nature of these themes—and how they differed across countries and settings—requires reconciliation of differences between their clinical importance, their impact at the population and public health level, and their importance to an individual woman's life. The fact that the implications diverge at each level should not mean that one is necessarily prioritized over another; MMWG members were compelled to acknowledge that many of their own preconceived assumptions about maternal morbidity were inadequate and/or incomplete. During the course of the group's collective work over five years, the conceptualization of maternal health evolved considerably, ultimately requiring a reframing of maternal morbidity.

This Supplement presents a series of papers sharing different aspects of the MMWG's work over the 5 years from 2012 to 2017, describing the evolution and paradigm shift in assessment of maternal morbidity to reflect women's lived experiences of it and the events related to it (pregnancy and childbirth), building on the evidence synthesized and elaborated by the group during that time. This series provides analysis and insight into the current state of evidence on maternal morbidity, and reports findings from the group's pilot test of the MMWG tool for measuring maternal morbidity. Taken together, the group of papers provides a synthesized and holistic view of maternal health, with implicit reference throughout to the underlying intellectual and academic question: “what does maternal morbidity mean?”

In describing a “new conceptual framework” for maternal morbidity (see Filippi et al. in this Supplement^4^), the MMWG reflects and elaborates on six key principles that form its foundation—first and foremost, the importance of using a woman‐centered approach. The updated maternal morbidity framework illustrates the broad ramifications of maternal morbidity and highlights the type of measurement that should take place to capture everything that matters to women, healthcare providers, and policy makers. The framework is also expected to have important implications for healthcare interventions and programs (see Firoz et al. in this Supplement^5^).

Ending preventable maternal mortality remains relevant and fundamental to achieving global development goals.^6^ Embracing the human‐rights‐based approach, all women, everywhere, need to receive the same level of high‐quality care before pregnancy and during pregnancy, labor, childbirth, and the postpartum period; the current reality falls short of this, and the risk of death remains tragically high. However, it is also imperative to expand the myopic focus on mortality to include morbidity, and to broaden the medicalized perspective—which focuses on clinical complications—to include the lived experiences of women. This is central to the theme of the Sustainable Development Goals (SDGs), which aspire to look beyond survival to health, empowerment, and well‐being. The MMWG's decision to move further beyond the focus on survival when thinking about morbidity (i.e. to move beyond only looking at cases of maternal near miss or severe morbidity) reflected the need to expand the clinical view of pregnancy. Once considered as “soft” topics, the findings of the MMWG highlight the critical need to reconcile the triad of the woman's perspective, the clinical/medicalized view of pregnancy, and public health priorities. Success in one area can only bolster the response in the other points of the triangle.

The work of the MMWG, from its analysis of the literature to the development and piloting of measurement tools, underlines the need for further research on maternal morbidities to be undertaken using mixed methods—both qualitative and quantitative—to close the vast gaps in knowledge on clinical conditions related to pregnancy and childbirth and the effects of social determinants and environmental factors. Consistent with the initiatives to place patients’ needs at the center of clinical care across the globe,^7^ maintaining a woman‐focused emphasis within approaches to measure and manage maternal morbidities is expected to improve the implementation of maternal health programs, in line with revised recommendations on the provision of maternal health care,^8^, ^9^ and revised targets and priorities, and, ultimately, to improve the lives of all women.

There is an urgent need to communicate the new conceptual framework on maternal morbidity and translate it for use by healthcare providers, academics, and decision makers. In order to “mainstream” the identification and management of maternal morbidity, the MMWG recognizes the need for continued refinement and development of the framework and related tools. Prior to global scale‐up, additional empirical research, peer review, and implementation activities are needed to guide efficient, evidence‐based, and sustainable roll‐out. To achieve this, the mantra of “health, empowerment, and well‐being” must be embedded in the daily lives of all women. The simplicity of the message belies the seriousness of the rallying call to bring attention to the urgent, unmet needs of women, their families, and communities. The findings of the MMWG clearly show that the ability to survive and thrive, and to participate productively in transforming society and the world, is not a privilege to be enjoyed by the few. Just as prevention of maternal mortality supports the human right to life,^10^ if the global community is to have a meaningful impact on maternal health then the reduction of maternal morbidity must also be recognized as a basic right.

## AUTHOR CONTRIBUTIONS

LS and DC conceptualized and steered the work from its initial development, and drafted and finalized the manuscript.

## MMWG MEMBERS

Kelli Barbour, Maria Barreix, Jose G. Cecatti, Maria L. Costa, Sara Cottler, Olubukola Fawole, Veronique Filippi, Tabassum Firoz, Luis Gadama, Atf Ghérissi, Gathari N. Gichuhi, Gill Gyte, Michelle Hindin, Anoma Jayathilaka, Amanda Kalamar, Yacouba Kone, Nenad Kostanjsek, Isabelle Lange, Laura A. Magee, Arvind Mathur, Affette McCaw‐Binns, Mark Morgan, Stephen Munjanja, Max Petzold, Elizabeth Sullivan, Frank Taulo, Özge Tunçalp, Rachel Vanderkruik, Peter von Dadelszen.

## CONFLICTS OF INTEREST

The authors have no conflicts of interest.
